# Prenatal Exposure to Organohalogens, Including Brominated Flame Retardants, Influences Motor, Cognitive, and Behavioral Performance at School Age

**DOI:** 10.1289/ehp.0901015

**Published:** 2009-08-31

**Authors:** Elise Roze, Lisethe Meijer, Attie Bakker, Koenraad N.J.A. Van Braeckel, Pieter J.J. Sauer, Arend F. Bos

**Affiliations:** 1 Division of Neonatology, Beatrix Children’s Hospital, University Medical Center Groningen and; 2 Beatrix Children’s Hospital, University Medical Center Groningen, University of Groningen, the Netherlands

**Keywords:** behavior, cognition, hydroxylated polychlorinated biphenyls, motor performance, neurotoxicity, organohalogens, pesticides, polybrominated diphenyl ethers, polychlorinated biphenyls, prenatal exposure, thyroid hormones

## Abstract

**Background:**

Organohalogen compounds (OHCs) are known to have neurotoxic effects on the developing brain.

**Objective:**

We investigated the influence of prenatal exposure to OHCs, including brominated flame retardants, on motor, cognitive, and behavioral outcome in healthy children of school age.

**Methods:**

This study was part of the prospective Groningen infant COMPARE (Comparison of Exposure-Effect Pathways to Improve the Assessment of Human Health Risks of Complex Environmental Mixtures of Organohalogens) study. It included 62 children in whose mothers the following compounds had been determined in the 35th week of pregnancy: 2,2′-bis-(4 chlorophenyl)-1,1′-dichloroethene, pentachlorophenol (PCP), polychlorinated biphenyl congener 153 (PCB-153), 4-hydroxy-2,3,3′,4′,5-pentachlorobiphenyl (4OH-CB-107), 4OH-CB-146, 4OH-CB-187, 2,2′,4,4′-tetrabromodiphenyl ether (BDE-47), BDE-99, BDE-100, BDE-153, BDE-154, and hexabromocyclododecane. Thyroid hormones were determined in umbilical cord blood. When the children were 5–6 years of age, we assessed their neuropsychological functioning: motor performance (coordination, fine motor skills), cognition (intelligence, visual perception, visuomotor integration, inhibitory control, verbal memory, and attention), and behavior.

**Results:**

Brominated flame retardants correlated with worse fine manipulative abilities, worse attention, better coordination, better visual perception, and better behavior. Chlorinated OHCs correlated with less choreiform dyskinesia. Hydroxylated polychlorinated biphenyls correlated with worse fine manipulative abilities, better attention, and better visual perception. The wood protective agent (PCP) correlated with worse coordination, less sensory integrity, worse attention, and worse visuomotor integration.

**Conclusions:**

Our results demonstrate for the first time that transplacental transfer of polybrominated flame retardants is associated with the development of children at school age. Because of the widespread use of these compounds, especially in the United States, where concentrations in the environment are four times higher than in Europe, these results cause serious concern.

Organohalogen compounds (OHCs) are toxic environmental pollutants used extensively in pesticides, flame retardants, hydraulic fluids, and in other industrial applications (Mariussen and Fonnum 2006). They are ubiquitously present in the environment, both in neutral and in phenolic form (Law et al. 2003). OHCs are known to bioaccumulate because of their high lipophilicity and resistance to degradation processes (Rahman et al. 2001) and have been detected in human adipose tissue and blood (Jensen 1987). In pregnant women these compounds are transferred across the placenta to the fetus (Lanting et al. 1998; Meijer et al. 2008). During this critical period of fetal growth and development, there is a risk for damage of the central nervous system because OHCs may interfere with developmental processes in the brain. Some compounds have effects on neuronal and glial cell development and are associated with disruption of neurotransmitters. Others interfere with endocrine systems, such as thyroid and sex hormones (Solomon and Schettler 2000; Weisglas-Kuperus 1998). OHCs may also produce their toxic effects through other pathways that are currently not well understood.

Previous studies in humans on the effect of prenatal OHC exposure on outcome reported that polychlorinated biphenyls (PCBs) have adverse effects on neurologic performance and cognitive development at 6–11 years of age (Boersma and Lanting 2000; Chen et al. 1992; Jacobson and Jacobson 1996; Stewart et al. 2008; Vreugdenhil et al. 2002). Knowledge of the neurotoxicity of PCBs led to their abandonment in most Western countries in the late 1970s. Despite this, metabolites of PCBs, the hydroxylated PCBs (OH-PCBs), are still present in high concentrations in maternal serum (Guvenius et al. 2003; Meijer et al. 2008). Previous studies postulated that OH-PCBs are even more toxic to brain development than are PCBs (Kimura-Kuroda et al. 2007; Kitamura et al. 2005). The long-term effect of prenatal OH-PCB exposure on human development is unknown.

Brominated flame retardants such as polybrominated biphenyls (PBBs) and polybrominated diphenyl ethers (PBDEs) were introduced as the new, allegedly harmless, successors of PCBs. However, the effect of prenatal exposure to brominated flame retardants on neurodevelopmental outcome at school age has never been investigated.

The primary aim of this explorative study was to investigate the influence of prenatal OHC exposure, including OH-PCBs and PBDEs, on motor, cognitive, and behavioral outcomes in healthy Dutch children at 5–6 years of age.

OHCs are also known to influence fetal thyroid hormone levels (Zoeller 2007). Because thyroid hormones are involved in neurodevelopmental processes, our second aim was to investigate whether thyroid hormone levels at birth were related to outcome in these children.

## Materials and Methods

### Cohort selection and sampling

This prospective cohort study is part of the Groningen infant COMPARE (Comparison of Exposure-Effect Pathways to Improve the Assessment of Human Health Risks of Complex Environmental Mixtures of Organohalogens) (GIC) study launched within the European COMPARE study. The cohort of the GIC study consisted of 90 white, healthy pregnant women randomly selected from those who had given birth to a healthy, full-term, singleton infant and lived in the northern provinces of the Netherlands (Meijer et al. 2008). All the women who had registered with midwives between October 2001 and November 2002 in the province of Groningen were invited to participate in the study.

To determine the concentrations of the neutral and phenolic OHCs, blood (30 mL) was taken from the women at the 35th week of pregnancy. The blood was centrifuged at 3,600 rpm for 10 min, and the serum was collected and stored in acetone-prewashed glass tubes at −20°C until analysis.

### Chemical analyses

Chlorinated OHCs [PCB-153 and 2,2′-bis-(4 chlorophenyl)-1,1′-dichloroethene (4,4′-DDE)], OH-PCBs (4OH-CB-107, 4OH-CB-146, and 4OH-CB-187), and a wood protective agent, pentachlorophenol (PCP), were analyzed in 90 serum samples taken at the 35th week of pregnancy. Because of financial constraints, brominated flame retardants [BDE-47, BDE-99, BDE-100, BDE-153, BDE-154, and hexabromocyclododecane (HBCDD)] were analyzed in 69 randomly selected serum samples taken at the 35th week of pregnancy. Mean levels of BDEs 47, 99, and 100 measured in blank samples were subtracted from values measured in study samples to correct for background exposures (4.8, 1.9, and 0.8 pg/g serum, respectively). Samples that were below the limit of detection (LOD) for BDE-47 (*n* = 2), BDE-99 (*n* = 3), or BDE-100 (*n* = 3) [0.08–0.16 pg/g serum (Meijer et al. 2008)]were assigned a concentration of 0 for analyses. Chemical and lipid analyses were performed as described elsewhere (Meijer et al. 2008).

### Thyroid hormone analyses

Thyroxin (T_4_), free T_4_, reverse triiodothyronin (rT_3_), triiodothyronin (T_3_), thyroid-stimulating hormone (TSH), and thyroid-binding globulin levels were determined in the umbilical cord blood of the 90 women, provided that enough cord blood was available to perform the analyses.

### Follow-up

We intended to include the 69 children for whom all the neutral and phenolic OHC concentrations had been determined. The children were invited prospectively to participate in an extensive follow-up program that assessed motor performance, cognition, and behavior at 5–6 years of age. Parents gave their informed consent for themselves and their children to participate in the follow-up program before the study. The study was approved by the Medical Ethical Committee of the University Medical Center Groningen and complied with all applicable international regulations.

### Motor outcome

To determine the children’s motor outcomes, we administered the Movement ABC, a standardized test of motor skills for children 4–12 years of age (Smits-Engelsman 1998). This test, which is widely used in practice and in research, yields a score for total movement performance based on separate scores for manual dexterity (fine motor skills), ball skills, and static and dynamic balance (coordination). Items on the Movement ABC included, for example, posting coins in a bank box, drawing a line between two existing lines of a figure, catching a bean bag, and jumping over a rope. The test required 20–30 min to administer. The tasks that make up the Movement ABC are representative of the motor skills that are required of children attending elementary school and are adapted to the children’s ages.

Supplementary to the Movement ABC, we assessed qualitative aspects of coordination and balance and fine manipulative abilities and the presence of choreiform dyskinesia, associated movements, sensory integrity, and tremors with Touwen’s age-specific neurologic examination (Touwen 1979). Approximately 20–30% of children from the general population obtain nonoptimal scores on one or two clusters of neurologic functions on Touwen’s neurologic examination. If a child’s score is nonoptimal on a specific item of the examination, the total score can still be within the normal range (Hadders-Algra 2002; Peters et al. 2008).

Finally, we administered the Dutch version of the Developmental Coordination Disorder Questionnaire (DCD-Q) (Schoemaker et al. 2006). This questionnaire, which is filled out by the parents, was developed to identify motor problems in children ≥ 4 years of age. It contains 17 items relating to motor coordination, which are classified into three categories: control during movement, fine motor skills/writing, and general coordination.

### Cognitive outcome

Total, Verbal, and Performance Intelligence levels were assessed using a short form of the Wechsler Preschool and Primary Scale of Intelligence, revised (WPPSI-R) (van der Steene and Bos 1997). Examples on items of the WPPSI-R are vocabulary, picture completion, and reproduction of block designs.

In addition, we assessed several neuropsychological functions to investigate whether these were impaired by prenatal OHC exposure. They were assessed by subtests of the NEPSY-II (Neuropsychological Assessment, 2nd ed.), a neuropsychological battery for children (Korkman et al. 2007). Central visual perception was assessed using the “geometric puzzles” subtest, in which the child is asked to match two shapes outside a grid with shapes inside the grid. Visuomotor integration was assessed by the “design copying” subtest, in which the child is asked to reproduce geometric forms of increasing complexity. Visuomotor integration involves the integration of visual information with finger–hand movements. Furthermore, we assessed inhibitory control with the “inhibition” subtest, which assesses the inhibitory control of automated behavior. In the first timed task, the child is asked to name a set of figures (i.e., squares and circles); in the second timed task, the child is asked to name the opposite of what is shown (i.e., squares instead of circles and circles instead of squares).

We assessed verbal memory using a standardized Dutch version of the Rey’s Auditory Verbal Learning Test (AVLT) (van den Burg and Kingma 1999). This test consists of five learning trials with immediate recall of words (tested after each presentation), a delayed recall trial, and a delayed recognition trial (van den Burg and Kingma 1999).

We measured sustained attention and selective attention with the two subtests “Score!” and “Sky Search” of the Test of Everyday Attention for Children (Manly et al. 2001). Sustained attention involves maintaining attention over an extended period of time. Selective attention refers to the ability to select target information from an array of distractors (Heaton et al. 2001). For example, the children were asked to count tones in 10 items, varying from 9 to 15 tones per item.

The total duration of the follow-up was approximately 2.5 hr. Test scores obtained when a child was too tired and uncooperative, as assessed by the experimenter, were excluded.

### Behavioral outcome

To obtain information on the children’s competencies and their behavioral and emotional problems, the parents completed the Child Behavior Checklist (CBCL) (Achenbach and Rescorla 2000) and the teachers filled out the Teacher’s Report Form (Achenbach and Rescorla 2000). These questionnaires consist of a total scale and two subscales: internalizing problems (emotionally reactive, anxious/depressed scales, somatic complaints, withdrawn behavior) and externalizing problems (attention problems and aggressive behavior).

In addition, the parents filled out an attention deficit/hyperactivity disorder (ADHD) questionnaire that contains 18 items on inattention, hyperactivity, and impulsivity (Scholte and van der Ploeg 2004).

To gain insight in the socioeconomic status (SES) and home environmental factors that may influence development, the highest level of maternal education and the Home Observation for Measurement of the Environment (HOME) questionnaire were assessed during the first year after birth during an earlier stage of the GIC study (Meijer et al. 2008).

### Statistical analyses

Chemical values are presented as medians with range because of the skewed distribution. Neutral compounds are expressed on lipid weight basis (nanograms per gram lipid) and phenolic compounds on fresh weight basis (picograms per gram serum). To compare the scores on the Movement ABC and cognitive tests with the reference values, we classified the scores into “normal” (> 15th percentile), “subclinical” (5th to 15th percentile), and “clinical” (≤ 5th percentile). We classified the questionnaires according to the instructions in the manual that provides the percentiles corresponding to the raw scores. The results on the neurologic examination are reported as percentage of children with nonoptimal function. We calculated intelligence quotient (IQ) scores by deriving the standard scores from the mean of the scores on the verbal and performance subtests. Because no Dutch norms are available for the NEPSY-II, we used the American norms to classify the scores of the children into percentiles. For the AVLT, we used the Dutch norms for children of 6 years of age. The Kolmogorov–Smirnov test was used to determine which neutral and phenolic OHC concentrations and outcome measures were distributed normally. We used the Pearson correlation for normally distributed variables and the Spearman’s rank correlation for nonnormally distributed variables, to relate the OHC concentrations to motor, cognitive, and behavioral outcome. The raw scores of the outcome variables were used for these calculations. Where appropriate, the test scores were inversely transformed so that for all tests higher scores indicated better outcomes. We used the Mann–Whitney *U*-test to relate the neurologic outcome (normal or abnormal) to OHC concentrations.

We corrected cognition and behavior of the children for SES and HOME, because these factors may exert an influence on the cognition and behavior of the children (Tong et al. 2007). We also investigated whether sex influenced the outcome measures in our study group (Mann–Whitney *U*-test). If so, we corrected for sex on that outcome measure. The corrections were performed by means of partial correlations controlling for confounders.

When correlations between OHCs and outcome did not reach significance, we explored their relationship by means of scatterplots, to determine whether some other, nonlinear relationship existed.

In this article, negative correlations indicate that higher OHC concentrations were related to worse outcome and positive correlations indicate that higher OHC concentrations were related to better outcome. Throughout the analyses, *p* < 0.05 was considered to be statistically significant. SPSS 14.0 software for Windows (SPSS Inc, Chicago, IL, USA) was used for all the analyses.

## Results

Of the 69 children invited, 62 (90%) participated in the follow-up program. Six sets of parents declined the invitation to participate. One girl had to be excluded because she suffered severe cognitive impairment of unknown origin and therefore could not be tested. The OHC concentrations of the seven children not followed up were not different from those who did participate.

Table 1 shows the concentrations of the neutral and phenolic OHCs measured at the 35th week of pregnancy of the 62 mothers and the concentrations of the thyroid hormones in the umbilical cord blood of 51 mothers.

The mean maternal age was 32 years (range, 24–42 years). The highest level of maternal education was primary school for 4 mothers, secondary school for 30 mothers, and tertiary school for 28 mothers. The mean score on the HOME questionnaire was 33 (range, 24–37).

### Outcome at school age

The cohort consisted of 38 boys and 24 girls. The mean age at follow-up was 5 years 10 months (range, 5 years 8 months to 6 years 2 months). Table 2 presents an overview of the children’s motor, cognitive, and behavioral outcomes. We excluded the test scores of two children on inhibition and sustained attention and scores of one child on visual perception and verbal memory, because they were too tired and uncooperative to attend the assessment. Their OHC concentrations were not different from those who did participate.

The scores of the children were comparable to the reference values, except for selective attention, verbal memory, and internalizing and externalizing behavioral problems, on which the children obtained slightly worse scores compared with the reference values. The mean (± SD) for total IQ of the children was 103 ± 9 (range, 82–125); mean verbal IQ, 102 ± 9 (range, 83–130); and mean performance IQ, 103 ± 13 (range, 73–133).

According to the neurologic examination, we found that of the 62 children examined, 1 child (2%) had coordination problems, 2 children (3%) had mild tremors, 18 children (29%) had nonoptimal fine manipulative abilities, and 21 children (34%) had nonoptimal sensory integration.

### OHCs in relation to outcome

Table 3 shows the OHCs that were significantly related to motor, cognitive, and behavioral outcome, uncorrected for possible confounders. We found both positive and negative correlations between OHCs and outcome. Brominated flame retardants correlated with worse fine manipulative abilities, worse attention, better coordination, better visual perception, and better behavior. Chlorinated OHCs correlated with less choreiform dyskinesia. OH-PCBs correlated with worse fine manipulative abilities, better attention, and better visual perception. The wood protective agent PCP correlated with worse coordination, less sensory integrity, worse attention, and worse visuomotor integration.

We corrected the cognitive and behavioral outcome for SES and HOME, and because boys and girls differed significantly for selective attention (*p* = 0.044), we corrected selective attention for sex. After these corrections, we found additional correlations between OHCs and outcome. Some correlations before the correction were stronger after controlling for confounders, whereas others disappeared. Table 4 presents these results and gives an overview of the number of analyses performed, including the correlations that nearly reached significance (*p* < 0.10).

Scatterplots of the relations between OHCs and outcome that did not reach significance revealed no further information about the existence of nonlinear relationships between variables (data not shown).

### Thyroid hormone analyses

Table 5 shows the thyroid hormones from the umbilical cord blood that were related to outcome at 5–6 years of age. TSH correlated with worse motor skills and worse attention. rT_3_ correlated with better fine manipulative abilities. T_3_ correlated with better visuomotor integration and better behavior. T_4_ correlated with better sensory integrity and less ADHD.

We also found that OHC concentrations were related to thyroid hormones. PCP correlated with lower concentrations of T_3_ (*r* = −0.292, *p* = 0.037); BDE-47 correlated with higher concentrations of T_3_ (*r* = 0.322, *p* = 0.021), as did BDE-99 (*r* = 0.311, *p* = 0.031) and BDE-100 (*r* = 0.291, *p* = 0.038).

## Discussion

The present explorative study indicated that prenatal background exposure to OHCs, including OH-PCBs and the more recently introduced PBDEs, correlated both positively and negatively with neurodevelopmental outcome in healthy Dutch children at 5–6 years of age. To the best of our knowledge, this study is the first to investigate the influence of background exposure to these toxic environmental pollutants on developmental outcome in healthy children at school age.

With regard to PBDEs, animal studies have indicated that prenatal exposure to different PBDEs may cause long-lasting behavioral alterations, particularly in motor activity and cognitive behavior (Gee and Moser 2008; Viberg et al. 2003). We found that brominated flame retardants also correlated with motor function, cognition, and behavior in humans. A study by Fischer et al. (2008) showed that BDE-99 has effects on behavior in mice. They found that BDE-99 and methylmercury exposure leads to disrupted spontaneous behavior. These results are in line with our findings in children that BDE-99 correlated with behavior, as measured with the CBCL.

Human studies on the 1974 Michigan PBB incident showed that accidental exposure to high levels of PBBs may lead to perceptual and perceptual–motor problems and lower scores on subtests of the McCarthy Scales of Children’s Abilities in children 4–6 years of age (Schwartz and Rae 1983; Seagull 1983). During this incident, Michigan residents unknowingly ingested PBBs through eggs, meat, and dairy products from animals whose feed had been inadvertently contaminated through the substitution of a fire retardant for a cattle feed supplement (Seagull 1983). Our study demonstrated that even background levels of brominated flame retardants exert an influence on diverse neurologic and neuropsychological functions in children at school age.

With regard to PCBs, exposure can lead to subtle cognitive deficits, motor delay, and adverse effects on neurologic status in children at school age (Boersma and Lanting 2000; Vreugdenhil et al. 2002). These effects, however, are often counteracted by the home environment (Vreugdenhil et al. 2002). Furthermore, Lee et al. (2007) described associations between chlorinated persistent organic pollutants and attention deficit disorder in children 12–15 years of age. Less is known about metabolites of PCBs. Recently, new techniques have become available to detect these metabolites in human serum. We found that, after correction for SES and the home environment, OH-PCBs correlated with multiple neuropsychological functions at school age: fine manipulative abilities, choreiform dyskinesia, verbal memory, inhibition, and behavior. Our results indicate that OH-PCBs might even be more neurotoxic than PCBs, as postulated in animal studies (Kimura-Kuroda et al. 2007; Kitamura et al. 2005).

OHCs are known to exert their neurotoxic influence by affecting thyroid hormone homeostasis. It is hypothesized that OHCs affect thyroid hormone homeostasis by interfering with thyroid hormone signaling in the developing brain, by changing intracellular thyroid hormone availability, and by interacting directly at the level of the thyroid hormone receptors. On the one hand, OHCs have a high affinity for thyroid hormone receptors and lead to a decrease in thyroid hormone levels, whereas levels of TSH increase through hormonal feedback mechanisms. Previous studies on pregnant women and their infants found that PCBs are associated with higher levels of TSH and lower levels of T_4_ (Koopman-Esseboom et al. 1994). We found that PCP correlated with lower levels of thyroid hormone but brominated flame retardants correlated with higher levels of thyroid hormone. It is unknown whether the underlying mechanism by which PCBs affect thyroid hormones is the same for these OHCs. Our study disclosed consistent relations between thyroid hormones and outcome. We found that TSH correlated with worse neuropsychological functions. Thyroid hormones (T_3_ and T_4_), by contrast, correlated with better outcome. These findings, together with the negative correlations between OHCs and development, seem to confirm the hypothesis that thyroid hormone homeostasis may be involved.

Because the threshold levels of toxicity for the different OHCs are unknown, we did not statistically test low versus high levels of OHCs in relation to outcome. The toxic equivalents of most of the OHCs investigated are also unknown. Research has shown that some compounds enhance each other, whereas others counteract each other. No data are available for all the compounds tested in our study. As a consequence, we explored relations between OHCs and outcome at school age by means of correlations. Because this study is, to the best of our knowledge, the first to investigate the association between background levels of OHCs and outcome at school age, it was difficult to hypothesize on the expected effect on the outcome measures. Our study might serve as a basis for power calculations for future studies, because it demonstrates the variability of various parameters and their mutual associations.

It was striking that we found both positive and negative correlations between OHCs and outcome. It is difficult to determine the implications of these results for functioning in later life. The multiple statistical analyses that were performed might have played a role in this finding. Furthermore, it is difficult to determine how many of these effects can reliably be assigned to the specific contaminants, because some of them were likely to show some degree of collinearity. Other contaminants that were not measured, such as methyl mercury, might also have played a role.

We did not compare postnatal OHC concentrations with outcome at 5–6 years of age because previous studies have pointed out that the most serious effects of neurotoxic OHCs are produced on developmental processes that occur prenatally (Jacobson et al. 1990; Vreugdenhil et al. 2002).

Concentrations of PBDEs in the environment are much higher in the United States than in Europe, Asia, or Australia (Costa and Giordano 2007; Rahman et al. 2001). Levels of PBDEs in breast milk have been increasing in the past 20–30 years, along with the serum levels in the general population worldwide (Costa and Giordano 2007; Jensen 1987; Sjodin et al. 2004). The fact that even low background levels of OHCs, as is the case in the Netherlands, may interfere with developmental processes, as illustrated by the present study, indicates that there is an urgent need for further research. We believe that future research should focus on longitudinal follow-up of children exposed to OHCs and should investigate the effect and nature of specific OHCs. Besides, the effect of combinations of OHCs (by means of composite scores) on neurodevelopment in large cohorts should also be investigated.

## Conclusions

Prenatal background exposure to OHCs not previously studied correlated with neuropsychological functioning in children at school age. PBDEs, used extensively worldwide, correlated with motor performance, attention, visual perception, and behavior, and we found the same results for OH-PCBs. We believe that unrelenting efforts should be made to find safe alternatives for these compounds.

## Figures and Tables

**Table 1:** OHC concentrations and thyroid hormone levels [median (range)].

Compound, medium	Concentration
OHC, maternal serum (*n* = 62)	
4,4′-DDE^a^	94.7 (17.5–323.8)
PCB-153^a^	63.0 (34.0–162.2)
BDE-47^a^	0.9 (< LOD–6.1)
BDE-99^a^	0.2 (< LOD–2.1)
BDE-100^a^	0.2 (< LOD–1.4)
BDE-153^a^	1.6 (0.3–19.7)
BDE-154^a^	0.5 (0.1–3.5)
HBCDD^a^	0.8 (0.3–7.5)
PCP^b^	1,018 (297–8,532)
4OH-CB-107^b^	26.0 (5.4–102.3)
4OH-CB-146^b^	103.3 (36.3–290.1)
4OH-CB-187^b^	79.3 (35.8–180.5)
Thyroid hormone, umbilical cord serum (*n* = 51)	
Free T_4_^c^	19.2 (12.0–25.1)
T_4_^d^	122 (76–157)
rT_3_^d^	3.9 (1.8–6.8)
T_3_^d^	0.8 (0.5–1.8)
TSH^d^	8.5 (3.5–23.5)
Thyroid-binding globulin^e^	30.5 (20.1–43.4)

LOD, limit of detection: 0.08–0.16 pg/g serum (Meijer et al. 2008).

a On lipid-weight basis (ng/g lipid).

b On fresh-weight basis (pg/g serum).

c In pmol/L.

d In nmol/L.

e In mg/L.

**Table 2:** Motor, cognitive, and behavioral outcomes [no. (%)].

Outcome	Normal^a^	Subclinical^a^	Clinical^a^
Motor outcome			
Movement ABC (*n* = 62)	55 (89)	4 (6)	3 (5)
DCD-Q (*n* = 62)	59 (95)		3 (5)
Cognitive outcome			
Total intelligence (*n* = 62)	60 (97)	2 (3)	
Verbal intelligence (*n* = 62)	60 (97)	2 (3)	
Performance intelligence (*n* = 62)	56 (90)	6 (10)	
Visual perception (*n* = 61)	60 (98)	1 (2)	
Visuomotor integration (*n* = 62)	60 (97)	2 (3)	
Verbal memory (*n* = 61)	44 (72)	14 (23)	3 (5)
Inhibition (*n* = 60)	52 (87)	7 (12)	1 (2)
Attention, sustained (*n* = 60)	54 (90)	6 (10)	
Attention, selective (*n* = 62)	44 (71)	11 (18)	7 (11)
Behavioral outcome			
Total behavioral problems^b^ (*n* = 62)	58 (94)	3 (5)	1 (2)
Internalizing problems^b^ (*n* = 62)	56 (90)	1 (2)	5 (9)
Externalizing problems^b^ (*n* = 62)	55 (89)	6 (10)	1 (2)
Total behavioral problems^c^ (*n* = 57)	51 (89)	4 (7)	2 (4)
Internalizing problems^c^ (*n* = 57)	51 (89)	4 (7)	2 (4)
Externalizing problems^c^ (*n* = 57)	52 (91)	3 (5)	2 (4)
ADHD questionnaire (*n* = 62)	57 (92)	2 (3)	3 (5)

a Normal was defined as > 15th percentile, subclinical as 5th to 15th percentile, and clinical as ≤ 5th percentile; with regard to intelligence, normal was defined as IQ > 85, subclinical as IQ 70–85, and clinical as IQ < 70.

b Derived from the CBCL (parents).

c Derived from the Teacher’s Report Form.

**Table 3:** OHCs in relation to outcome.

OHC	Function	Correlation coefficient (*R*)^a^	*p*-Value
Brominated flame retardants			
BDE-47	Attention, sustained	−0.267	0.039
	Internalizing behavior^b^	0.301	0.018
	Total behavioral outcome^b^	0.288	0.024
	Coordination^c^	0.255	0.045
BDE-99	Internalizing behavior^b^	0.323	0.013
	Total behavioral outcome^b^	0.281	0.032
BDE-100	Coordination^c^	0.309	0.014
	Internalizing behavior^b^	0.403	0.001
	Externalizing behavior^b^	0.305	0.017
	Total behavioral outcome^b^	0.389	0.002
BDE-153	Visual perception	0.289	0.026
BDE-154	Fine manipulative abilities^c^	−0.300	0.018
HBCDD	Coordination^c^	0.290	0.023

Chlorinated OHCs			
PCB-153	Choreiform dyskinesia^c^	0.345	0.007
4OH-CB-107	Fine manipulative abilities^c^	−0.311	0.016
	Attention, selective	0.293	0.021
	Visual perception	0.278	0.030
4OH-CB-187	Attention, selective	0.318	0.012
4,4′-DDE	Choreiform dyskinesia^c^	0.308	0.016

Wood protective agent			
PCP	Coordination^c^	−0.363	0.004
	Sensory integrity^c^		0.034^d^
	Visuomotor integration	−0.287	0.024
	Attention, selective	−0.254	0.046

a Positive correlations indicate better outcome and negative correlations indicate worse outcome.

b Derived from the CBCL (parents).

c Derived from Touwen’s neurologic examination.

d Calculated by the Mann–Whitney *U*-test.

**Table 4:** Correlation coefficients for OHCs in relation to outcome, corrected for SES, HOME, and sex.

Outcome	BDE-47	BDE-99	BDE-100	BDE-153	BDE-154	HBCDD	PCB-153	4,4′-DDE	4OH-CB-107	4OH-CB-146	4OH-CB-187	PCP
Movement ABC												
Coordination^a^						0.290**	0.244*	0.239*				−0.363#

Fine manipulative abilities^a^					−0.253*				−0.311**			

Tremors^a^												

Sensory integration^a^												
Choreiform dyskinesia^a^							0.345#	0.308**		0.228*		

DCD-Q												
Total intelligence						0.393**						

Verbal intelligence						0.479#						

Performance intelligence								0.315*				−0.337**

Visual perception												−0.255*

Visuomotor integration												
Verbal memory				−0.723#								

Inhibition									−0.310*	−0.355**	−0.346**	

Attention, sustained	−0.264**	−0.264**	−0.261*									

Attention, selective	0.230*											0.439#

Total behavioral outcome^b^		0.276**	0.231*									

Internalizing behavior^b^		0.283**	0.253*					0.344**				

Externalizing behavior^b^										−0.278*		

Total behavioral outcome^c^							−0.314*			−0.411**		

Internalizing behavior^c^	0.237*	0.265*	0.236*							−0.298*		

Externalizing behavior^c^							−0.288*			−0.328**		

ADHD questionnaire												

a Derived from Touwen’s neurologic examination.

b Derived from the CBCL (parents).

c Derived from the Teacher’s Report Form.

* *p* < 0.10,

** *p* < 0.05,

# *p* < 0.01.

**Table 5:** Thyroid hormones in relation to outcome.

Thyroid hormone	Function	Correlation coefficient (*R*)^a^	*p*-Value
TSH	General motor skills^b^	−0.430	0.002
	Fine manipulative abilities	−0.291	0.038
	Attention, sustained	−0.298	0.038
rT_3_	Fine manipulative abilities	0.279	0.047
T_3_	Visuomotor integration	0.308	0.028
	Internalizing behavior^c^	0.319	0.031
T_4_	Sensory integrity		0.007^d^
	Attention deficit/hyperactivity	0.380	0.009

a Positive correlations indicate better outcome, and negative correlations indicate worse outcome.

b Assessed with the DCD-Q.

c Derived from the CBCL (parents).

d Calculated by the Mann–Whitney *U*-test.
